# Mobile Deep Learning System That Calculates UVI Using Illuminance Value of User’s Location

**DOI:** 10.3390/s21041227

**Published:** 2021-02-09

**Authors:** Seung-Taek Oh, Deog-Hyeon Ga, Jae-Hyun Lim

**Affiliations:** 1 Smart Natural Space Research Center, Kongju National University, Cheonan 31080, Korea; ost73@kongju.ac.kr; 2 Department of Computer Science & Engineering, Kongju National University, Cheonan 31080, Korea; bigstring@smail.kongju.ac.kr; 3 Department of Urban Systems Engineering, Kongju National University, Cheonan 31080, Korea

**Keywords:** mobile deep learning, *UVI*, illuminance, user’s location, natural light

## Abstract

Ultraviolet rays are closely related with human health and, recently, optimum exposure to the UV rays has been recommended, with growing importance being placed on correct UV information. However, many countries provide UV information services at a local level, which makes it impossible for individuals to acquire user-based, accurate UV information unless individuals operate UV measurement devices with expertise on the relevant field for interpretation of the measurement results. There is a limit in measuring ultraviolet rays’ information by the users at their respective locations. Research about how to utilize mobile devices such as smartphones to overcome such limitation is also lacking. This paper proposes a mobile deep learning system that calculates *UVI* based on the illuminance values at the user’s location obtained with mobile devices’ help. The proposed method analyzed the correlation between illuminance and *UVI* based on the natural light DB collected through the actual measurements, and the deep learning model’s data set was extracted. After the selection of the input variables to calculate the correct *UVI*, the deep learning model based on the TensorFlow set with the optimum number of layers and number of nodes was designed and implemented, and learning was executed via the data set. After the data set was converted to the mobile deep learning model to operate under the mobile environment, the converted data were loaded on the mobile device. The proposed method enabled providing UV information at the user’s location through a mobile device on which the illuminance sensors were loaded even in the environment without *UVI* measuring equipment. The comparison of the experiment results with the reference device (spectrometer) proved that the proposed method could provide UV information with an accuracy of 90–95% in the summers, as well as in winters.

## 1. Introduction

Ultraviolet rays (100–380 nm), which have a wavelength band shorter than visible light as part of sunlight, reach the ground surface and greatly affect human health [1]. In general, ultraviolet rays have been recognized to adversely affect the skin and the eyes, such as promoting skin aging, skin cancer, keratitis, and cataracts [2]. However, UV rays contribute absolutely to the synthesis of *vitamin D*, an essential component of human health and contribute to musculoskeletal system, cardiovascular disease, and mental health [3,4]. As these findings become known, in recent years, adequate UV exposure for health maintenance has been recommended, and the necessity and importance of accurate UV information has been emphasized [5,6]. Some studies have estimated the intensity of UV light according to the location of latitude and longitude, suggesting an appropriate UV exposure time [7]. Moreover, many countries provide the UV intensity and corresponding outdoor activity recommendation services through government agencies and organizations [8]. However, these services have been based on the results of UV measurements collected through regional base stations and were limited to UV information for a wide range of locations, and could not provide accurate UV information that could be changed according to the local characteristics and weather elements. Meanwhile, specialized optical measuring equipment such as spectrometers or ultraviolet sensing devices have been applied to obtain more accurate UV information. Herndon et al. confirmed and published about various UV wavelength bands (UVA, UVB, and UVC), which affected living organisms on the earth and reached the ground surface by measuring solar light through a spectral radiometer [9]. Banerjee et al. conducted an actual solar light measurement with the currently commercially available wearable UV measuring devices and UV Erythema Broadband Radiometer. They proposed that the best performing wearable UV measuring devices could measure the *UVI* with an error range within 20% compared to those with a UV radiometer [10]. However, there was a limit to the use of the general public due to the need for expertise in optical measurement and expensive equipment and sensing devices for UV measurement. Recently, technologies using sensors in the mobile device to check optical characteristics such as illumination and color temperature, which have been environmental factors around the user, or an individual’s health state have been generalizing [11,12]. Moreover, as the technology related to artificial neural network develops, research cases are being introduced to solve and predict complicated problems by establishing a deep learning model through the characteristic analysis between highly correlated factors [13,14]. Meanwhile, Mei et al. calculated the UV intensities through the images collected through smartphones’ CMOS sensors [15]. Feister et al. introduced a method based on the regression equation that calculated the UV information with the element values such as illuminance of solar light and solar zenith angle [16]. However, it could not be sufficiently validated for servicing the *UVI* and expected amount of *vitamin D* synthesis because the experiment was performed under the limited conditions of specific dates or climate conditions. Further, Fatma et al. have estimated the particulate matter (PM10) values by inputting the environmental factors such as nitrogen monoxide (NO) and ozone (O_3_) by the introduction of neural network technology [17]. Meanwhile, Jacovide et al. introduced a neural network model that calculated the solar UV and the photosynthetically active radiation (PAR) via the input of sunshine fraction, air temperature, and humidity [18]. However, until now, attempts to derive useful information such as the intensity of ultraviolet rays by analyzing the optical characteristics of sunlight, which were known to be relevant, or by applying deep learning techniques have been lacking.

Therefore, this paper proposed a mobile deep learning system that calculates the *UVI* through input the luminance at the user’s location. The proposed system consisted of a natural light database that supported data set extraction of the deep learning, a deep learning server that supported construction and learning of the artificial neuron network model, and a mobile device that measured the luminance at the user’s location and implements UV information service. In the deep learning server, a data set was constructed with the values of the luminance, *UVI*, and solar zenith angle through characteristics analysis of the natural light. The deep learning model that calculated the *UVI* via inputting the luminance, solar zenith angle, and monthly characteristics was designed and implemented, and then, the learning was executed by means of the learning data set. The learned model was then converted to the mobile deep learning model and the model was distributed, so that the mobile deep learning system that calculated the *UVI* through the illuminance data collected via the mobile device of the user was realized. Therefore, the method of providing UV information using the illuminance sensors in the mobile device and deep learning technology even without UV measuring equipment was proposed. It aimed to validate the *UVI* calculation performance of the proposed method by the comparison experiment with those of spectral radiometer and by calculating and comparing the expected amount of *vitamin D* synthesis drawn by the equation to confirm the potential provision of UV information to improve the user’s health.

## 2. Mobile Deep Learning System

**Figure 1:**
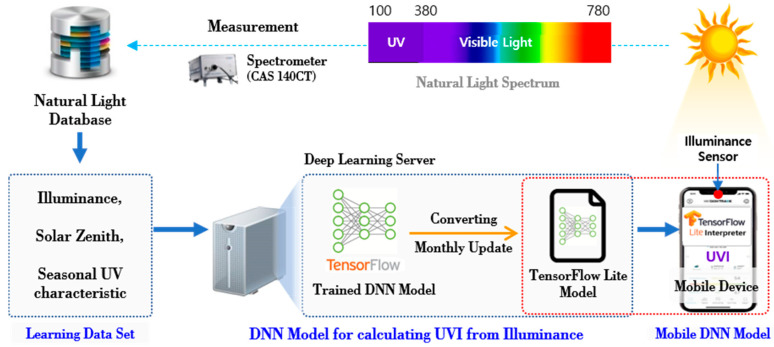
Overview of the mobile deep learning system.

In this paper, a mobile deep learning system that calculated the *UVI* through measurement of the luminance at the user’s location by means of mobile devices such as smart phones that were easily used by many peoples, was proposed. The proposed system was realized by the natural light DB collected through actual measurement, the learning data set extracted from the natural light DB, and the deep learning server and mobile device to establish the deep learning model and implementation. Figure 1 shows the overview of the proposed system.

The characteristics of the natural light were measured through the spectrometer (CAS 140CT, Instrument Systems, Munich, Germany) in order to develop the proposed system, and the measured data were stored in the natural light DB. The measurement was carried out at the location of latitude of 36.85° and the longitude of 127.15° for about 2 years and 6 months (April 2017–September 2019) in order to collect optical characteristics of irradiance, illumination, color temperature, and chromaticity coordinate by wavelength. The correlation between the characteristics of the natural light was analyzed, the input variables of the deep learning model were selected, and then the data set was extracted for the leaning. The deep learning model based on the TensorFlow (Open-source library, Google, Pasadena, CA, USA) was then designed and realized followed by execution of the learning through the data set extracted in the previous stage. The model was converted to the mobile deep learning model format (TensorFlow Light) to execute the model in the mobile device, and it was distributed to the mobile devices of the users. In the mobile device, the illumination was measured through the sensor, and the illumination data were inputted into the mobile deep learning model to calculate the *UVI* in order to provide the relevant UV information.

## 3. Natural Light DB-Based Learning Data Set

**Figure 2:**
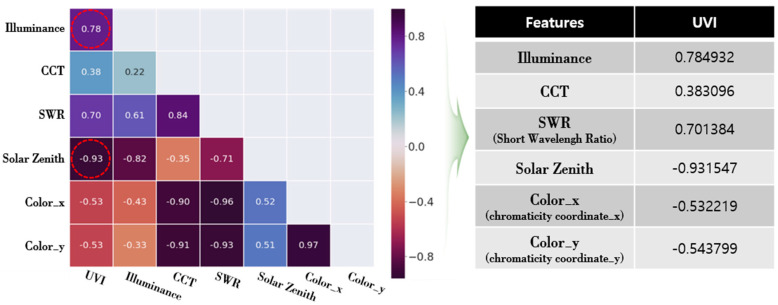
Correlation analysis results between the characteristics of the natural light.

In order to calculate the *UVI* through the proposed method, input variables of the deep learning model, which had a high correlation with the output variables and was easy to measure by the user, were selected, and data set for the model learning were needed [19]. For that, the correlation between the characteristics of the natural light was analyzed through the measured natural light DB. The correlations between illuminance, color temperature, chromaticity coordinates (x,y) which were judged possible to collect through the illuminance and color sensors that were loaded on most of the mobile devices and short wavelength ratio of visible light, and *UVI* were analyzed. In addition, the correlation between the *UVI* and the solar zenith angle that have been known to have a high correlation through the previous study cases was also examined [20]. The solar zenith angles, provided by the Korea Astronomy and Space Science Institute for the areas where the natural light was measured, were applied. Figure 2 shows the correlation between the characteristics of the natural light, the solar zenith angles, and the *UVI*.

**Figure 3:**
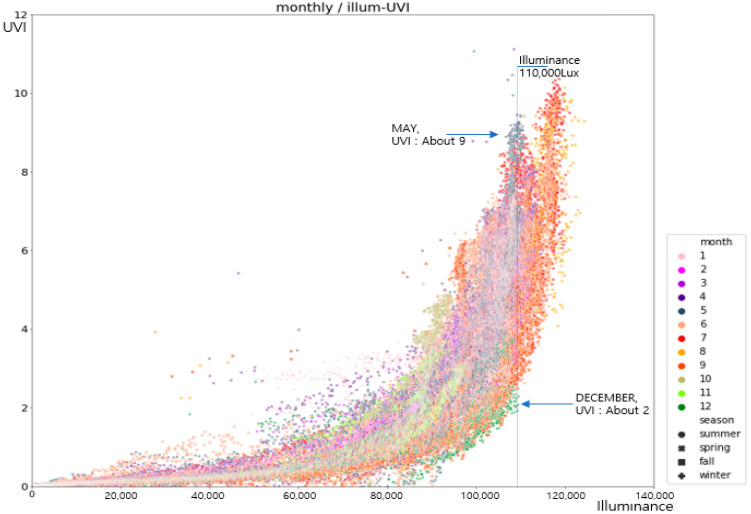
Monthly illumination-*UVI* (Ultraviolet Index) distribution characteristics.

The correlation between the characteristics of the sunlight through the analysis results in Figure 2 was the highest between the illumination and the *UVI* with about 0.766. The solar zenith angle, which was a variable obtained through the GPS sensor in the mobile device, was highly and negatively correlated with the *UVI* (correlation coefficient of −0.931). On the contrary, the chromaticity coordinates (x, y) of the natural light had a low correlation. The short wavelength ratio, though it had a high correlation, was judged as not suitable as a variable for the deep learning since there was a limit in data collection through the mobile devices. Furthermore, considering the previous study cases that said that natural light and the *UVI* could have different modes in reaching to the ground surface due to the seasons or monthly weather characteristics, the monthly illumination and the *UVI* distribution were analyzed. The results are illustrated in Figure 3 [21].

In Figure 3, the monthly illumination and *UVI* at the measurement location of the natural light (Cheonan, Korea) shows that as the illumination was increased, the *UVI* was also increased, in general. In the figure, the annual maximum illuminance was about 120,000 lux and the annual maximum *UVI* was 12 or higher. In addition, the *UVI* was higher during the relatively warm seasons (May–July), and it was low during the cold seasons (November–January). Specifically, the comparison results of the maximum *UVI* values in the same illuminance (110,000 lux) showed that the *UVI* was about 2 in December and it was about 9 in May, exhibiting a prominent difference of the *UVI* by month (season) even if the same illuminance was measured. The monthly maximum *UVI* values were extracted for 1:00 p.m. during the noon-afternoon where the UV index was expected to be the maximum and the correlation between the illuminance and the *UVI* was calculated according to Pearson’s correlation coefficient. The results are arranged in Table 1.

The monthly maximum illuminance was relatively low in January, October, November, and December, whereas it was high in June, July, and September. However, the *UVI* was higher than 10 only in June and July, and it was relatively low with about 8.3 in September even under the similar illuminance. The illuminance and the *UVI* were relatively low in August compared with the adjourning months, which was due to the high rainfall. Moreover, the correlation between the illuminance and the *UVI* was high about 0.85 in February, March, and November, whereas it was low with about 0.76 in August and September, which indicated that the distribution patterns of the illuminance and the *UVI* were different. It was anticipated that it would be advantageous to reflect monthly UV characteristics together to calculate the *UVI* through the illuminance values at the user’s location. The analysis results showed that the *UVI* was, in general, highly correlated with the illuminance of sunlight and solar zenith angle. Therefore, multiple linear regression or neural network model was assumed to be possibly applied in this study. Nonetheless, since the differences in correlation by month/time were large, the neural network model could predict more accurate results in the proposed method to which various elements with significant variations were inputted [22].

In addition, it was judged as beneficial to calculate an accurate *UVI* if the monthly UV characteristics were reflected in the mobile deep learning model. The data set for mobile deep learning was extracted through the *UVI*, illuminance, and solar zenith angle based on the correlation between the above characteristics of the natural light, monthly illuminances, and *UVI* patterns.

## 4. Mobile DNN Model for Calculation of the Illuminance-Based UV Information

**Figure 4:**
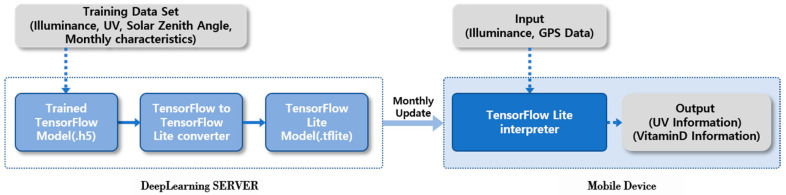
Construction and execution flow of the mobile deep learning model.

In the study, an artificial neuron network model, executed by linking the deep learning server and the mobile device, was realized in order to calculate UV information through input the illuminance values at the user’s location. An artificial neural network is a model that solves the problem while making the connection intensities of the neuron (node), which was composed of the input layer, hidden layer, and output layer adapted and changed to the optimum condition. The DNN was constructed with more than two hidden layers [23]. Deep learning technology has been utilized in computer vision, natural language processing, and voice recognition [24]. Saez et al. applied the deep learning technology in the recognition of activity [24], while Merenda et al. applied the mobile deep learning library that was provided by Google to realize the Edge Computing for IoT devices, all of which broadened the scope of the deep leaning [25]. The deep learning server was made to support implementation of the deep learning model and the mobile service through the natural light data set, while the mobile device would execute the deep learning model and provide the UV information service. Figure 4 shows the construction and the execution flow of the proposed mobile DNN (Deep Neural Network) model.

The deep learning model in Figure 4 shows the calculation results of the *UVI* intensity by means of input the illuminance data measured at the current location via illuminance sensor in the mobile device of the user as well as the GPS-based solar zenith angle information. In the deep learning server, the Keras (2.1.1) framework was used to realize the neural network model code, and the open-source library TensorFlow of the Google that has been widely used recently to predict changing atmospheric quality and weather observation data was applied [26,27]. The predication and calculation performance of the artificial neural network model based on the TensorFlow depends on the key setting variables such as each input, output, hidden layer, and number of nodes [28]. Therefore, a comparison experiment was carried out to select the key input parameter, hidden layer, and number of nodes necessary for implementation of the optimum artificial neuron network model. First, the variables of illuminance, solar zenith angle, and seasonal UV characteristics ratio were combined to decide the input variables; these variables were set as each input of the artificial neuron network model, and then, the performance of each model was examined. The performance of each model was compared by calculating mean absolute errors (MAE). In addition, the calculation result of each model was confirmed while increasing the number of the hidden layer from 1 to 3 to compare the difference according to the construction of the multiple hidden layers. The comparison results are displayed in Table 2. Since there was no standard provided for the number of the hidden layer, it was set to 16, which was a multiple of 2 closest to the number of input nodes (n) when the illuminance, solar zenith angle, and monthly characteristics were inputted according to the methods proposed in the existing literatures and researches [29].

**Figure 5:**
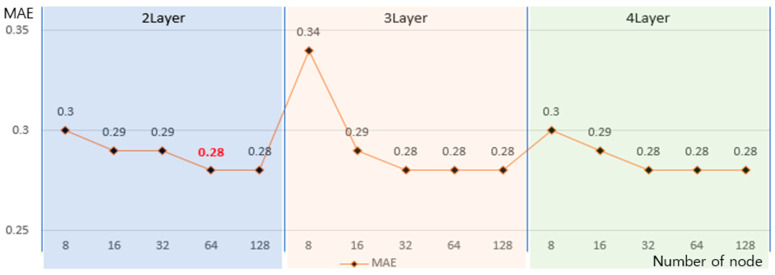
Experimental results for optimization of the Deep Neural Network (DNN) model.

In Table 2, the average MAE, when the illuminance, solar zenith angle, and monthly characteristics were used altogether as the input values, was the most excellent showing 0.30, whereas when the hidden layer was multiple in number under the same condition, the MAE was excellent with 0.29. The illuminance, solar zenith angle, and monthly characteristics were selected as input variables for the proposed DNN model based on the results in Table 2, and it was constructed with multiple hidden layers. One-Hot Encoding method was applied so that the monthly (January–December) characteristics could be processed as type data not as simple numeric data [30]. In addition, the number of the hidden layers and the number of nodes in each hidden layer were adjusted once again to calculate more accurate UV information. The results are illustrated in Figure 5.

**Figure 6:**
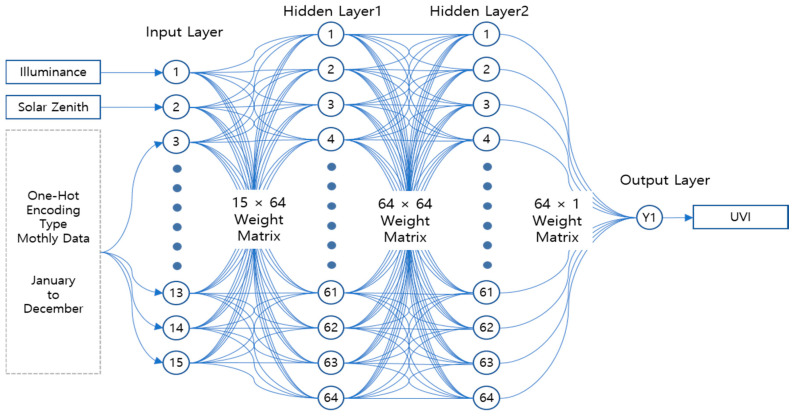
DNN model for the illumination-based *UVI* calculation.

Figure 5 shows the comparison results of the MAE in each stage while adjusting the number of the hidden layer to 2–4 and the number of the node to 8, 16, 32, 64, and 128. When the numbers of nodes were more than 64, more than 32, and more than 32 with the numbers of hidden layer of 2, 3, and 4, respectively, the best MAE of 0.28 could be obtained. Therefore, for the Deep Neural Network (DNN) that proposed to make the hidden layer and nodes minimum while maintaining the minimum MAE, the hidden layer was set to 2 and the number of the node in each hidden layer was set to 64. A learning that adjusted a connection process between each node of the DNN model by itself was conducted through the data set extracted from the analysis of three sheets of measured natural light DB. The activation function that converted the input signal to the output signal ReLU (Rectified Linear Unit) that has been used the most to process nonlinear data was applied [31]. Figure 6 shows the implementation results of the DNN model that calculated the illumination-based UV information.

The DNN model based on the TensorFlow was realized according to Figure 6, and it was converted to the deep model form TensorFlow Lite Model for the mobile device [32]. The converted deep learning model was distributed to the mobile devices of each user through the server to provide the UV information at the user’s location in real time regardless of internet connection status. However, a mobile DNN model that was renewed continuously according to the latest natural light DB by way of the monthly update function through the deep learning server. Moreover, in the user’s mobile device, the TensorFlow Lite Interpreter was installed together so that the distributed deep learning model could be interpreted and executed. The interpreter was able to provide the accurate *UVI* intensity at the user’s location. Moreover, the expected *vitamin D* synthesis information was also provided to support an adequate exposure to UV. The expected amount of *vitamin D* synthesis was calculated with Equation (1) proposed in the research by Godar [33].

(1) $$VitD\;\left[IU\right]=49\;VUD\left[\frac{J}{m^{2}}\right]\cdot \;\frac{STF\cdot PBE\cdot AF}{SPF}.$$

In Equation (1), the *VUD* was the *vitamin D* weighting UV rays’ amount, *STF* is the skin type factor where Type II has an *STF* value of 3.2/3, type III has 3.2/4, type IV has 3.2/5.25, and type V has 3.2/7.5 [33]. The percent body exposure (*PBE*) is the body exposure ratio, which is differently applied by the season and age [34], while the AF refers to the age factor having 1.0 for younger than 21, 0.83 between age 22 and 40, 0.66 for age 41–59, and 0.49 for age older than 60 [35]. *SPF* is the sun protection factor and is presented as values between 0 and 1, with 1 being no UV protection agent used. Equation (2) is the formula to calculate the accumulation amount (*DUV_out_*) by an hour of *vitamin D* UV irradiance by exposure to the sunlight. In order to calculate the UV intensity outdoors at the user’s location, *DUV* calculation equation (Equation (3)), which applied action spectrum conversion factor (*ASCF*) and geometric conversion factor (*GCF*), was used [36].

(2) $$VUD=\int_{0}^{t_{c}}DUV_{out}\left(t\right)\;dt$$

(3) $$DUV_{out}=EUV\left[W/m^{2}\right]\cdot ASCF\cdot GCF.$$

*ASCF* in Equation (3) is the correction factor of the action spectrum, which converts the UV irradiance reacting the horizon into the body surface’s radiance intensity. It is a variable considering the season and user’s location, which was introduced in the research by the Pope. The *ASCF* (1.049 for spring, 1.104 for summer, 1.029 for autumn, and 0.842 for winter) and *GCF* (0.600 for spring, 0.600 for summer, 0.655 for autumn, and 0.655 for winter), which corresponded to the latitude of 35° N, were applied in this study [37]. In addition, the *EUV* was the *UVI* where erythema weight was applied and calculated through Equations (4) and (5) [36].

(4) $$UVI=\;k\int_{280}^{400}E\left(\lambda \right)\cdot W\left(\lambda \right)\;d\lambda =\;k\;\left[m^{2}/W]\;\cdot \;EUV\;[W/m^{2}\right],$$

(5) $$EUV=\;UVI/k\;\left[\frac{W}{m^{2}}\right],\;\left(k=40\right).$$

In the Equation (4), *UVI* could be cumulatively calculated with the weightage according to the wavelength in the spectral irradiance in the 280–400 nm band, but in this paper, the *UVI* values calculated through the DNN model were used. The *EUV* was calculated through Equation (5) and the *UVI* as well as the amount of expected *vitamin D* synthesis for current illuminance were provided through the mobile device.

## 5. Performance Evaluation

**Figure 7:**
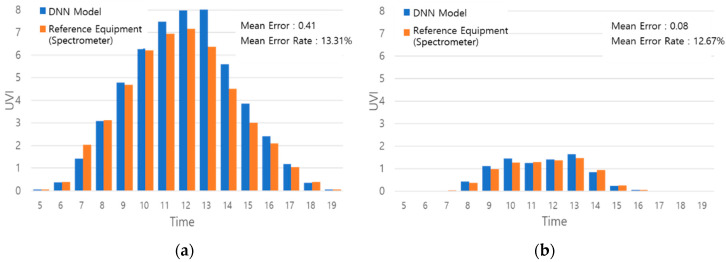
Experiment for *UVI* calculation performance of the mobile DNN model. (**a**) Summer(12 June 2019); (**b**) Winter(27 November 2019).

An experiment was conducted to evaluate the accuracy of illuminance-based *UVI* calculation method and the possibility of UV information service. The results of the *UVI* calculated through the proposed mobile DNN model after inputting the illuminance at the user’s location and measurement though spectrometer (CAS 140CT, Instrument Systems, Munich, Germany) were compared. The mobile device used was the smart phone from Company S (A5 2017, Samsung, Suwon, Korea) for the UV information service such as measurement of the illuminance and installation of the DNN model. The experiment was carried out on the day of the highest UV intensity and on the selected day in summers having the highest UV intensity and in winters having the lowest UV intensity. The experimental results are displayed in Figure 7.

The *UVI* calculated through the proposed method in Figure 7 showed errors of 0.41 in summers (Figure 7a) and 0.08 in winters (Figure 7b) compared with the measurement results through the reference device. A relatively high error was found in the summers (Figure 7a) where the *UVI* indices were high, while lower error was observed in the winters (Figure 7b) where the *UVI* indices were relatively low. The mean errors in each season considering the maximum *UVI* and variation of the *UVI* by season were 13.31% and 12.67%, respectively, showing an insignificant performance difference by season. In addition, the expected amounts of *vitamin D* synthesis during the expected activity hours (7:00 a.m.~4:00 p.m.) on each of the experimental day to check supporting performance of an adequate UV exposure were proposed. The results are tabulated in Table 3.

Table 3 shows the calculation results of the expected amount of *vitamin D* synthesis assuming that adults of age higher than 21 years with the skin type II in Korea (latitude: 36.85, longitude: 127.15) exposed to the solar *UVI* for 20 min (skin exposure area 15%, sun cream not used) under the *UVI* condition by each time. The amount of *vitamin D* synthesis during summers (12 June 2019) was on an average of 598.52 *IU* when the measurement value of the reference equipment was employed, whereas it was 660.11 *IU* by way of the calculation result of the proposed method. In addition, the amount of *vitamin D* synthesis in winters (27 November 2019) was on an average of 104.45 *IU* when the results of the reference equipment were employed, and it was 109.88 *IU* with the calculation results through the proposed method, which could provide the expected amount of *vitamin D* having an accuracy of about 90–95% compared with the values through the reference equipment.

## 6. Conclusions

As ultraviolet rays in the sunlight absolutely attribute human *vitamin D* synthesis, recently optimum exposure to UV has been recommended to maintain health, and consequently, the necessity of accurate UV information also has been increasing. In this paper, a mobile deep learning system was proposed to provide an accurate UV information through the illumination values at the users’ locations. Accordingly, the correlation between the characteristics of natural light such as the illumination and *UVI* was analyzed through the natural light DB measured and collected with a spectrometer for about 2 years and 5 months (April 2017–August 2019) at the latitude of 36.85° and longitude of 127.15° (Kongju National University, Cheonan, Korea), and then, a data set was extracted for the deep learning model. In addition, it was confirmed that there was a high correlation between the *UVI* and the solar zenith angle, and monthly distribution of the illumination and *UVI* was different. A data set comprised characteristics values of the illuminance, *UVI*, and solar zenith angle was extracted based on the characteristics analysis results of the natural light. A selection process of the input variables to realize an optimum deep learning model and selection process of number of the layer and nodes were performed. The deep learning was, therefore, designed to have two hidden layers that input the illumination, solar zenith angle, and monthly characteristics as well as 64 nodes in each hidden layer. Implementation of the deep learning model in the mobile deep learning server was followed through the TensorFlow, which was an open-source library of Google, and then, a learning based on the data set extracted through the analysis of the natural light DB was executed. The TensorFlow model (.h format) with the completed learning was converted to the TensorFlow Lite model (.tflite), and then, it was distributed to the mobile devices to execute the proposed system through the interpreter of the deep learning model. With these factors, a system technology based on deep mobile learning, which could provide UV information at each user’s location, was realized through the input of the illuminance collected through the mobile device even when a separate UV-measuring equipment was not equipped. The actual measurement was performed to compare the proposed model loaded on the smartphone (Galaxy S, Samsung, Korea) with the reference device (spectral radiometer, CAS 140CT). The results suggested that accurate *UVI* information could be calculated with the accuracies of 90% and 95% during summers and winters, respectively. Furthermore, the expected amount of *vitamin D* synthesis was also provided based on the calculated *UVI* information so that the UV information service could also be realized to improve users’ health using the mobile device.

In the future, efforts will be made on the improvement of *UVI* calculation accuracy through renewal of the learning data and continuous leaning of the mobile deep learning model. Furthermore, it is planned to conduct researches for development of the UV information system based on the flatform that can collect and share the UV information, which is highly related with the individual health, through mobiles used by most of the people.

## Figures and Tables

**Table 1:** Pearson’s correlation between the monthly average illuminances and the *UVI* (Ultraviolet Index).

Month	1	2	3	4	5	6	7	8	9	10	11	12
Illuminance	109,553	105,979	113,366	114,582	114,631	118,696	119,874	110,516	118,333	108,353	98,414	109,484
*UVI*	5.66275	3.70099	7.3346	8.35783	9.403	14.3897	10.3591	8.45462	8.32092	6.87911	3.42396	4.35982
Correlation	0.7908	0.8588	0.8951	0.8422	0.8368	0.8156	0.8230	0.7197	0.7595	0.7959	0.8449	0.7766

**Table 2:** Pearson’s correlation between the monthly average illuminances and the *UVI*.

Input Variables/Number of Hidden Layers	1	2	3
Illuminance	0.81	0.81	0.81
Illuminance and solar zenith angle	0.38	0.37	0.36
Illuminance, solar zenith angle, and monthly characteristics	0.31	0.29	0.29

**Table 3:** Comparison of expected amount of *vitamin D* synthesis with the *UVI* calculation results (12 June 2019–27 November 2019).

Input	Summer (12 June 2019)				Winter (27 November 2019)			
	Reference Equipment (CAS 140CT)		Proposed Model		Reference Equipment (CAS 140CT)		Proposed Model	
	*UVI*	*Vitamin D*	*UVI*	*Vitamin D*	*UVI*	*Vitamin D*	*UVI*	*Vitamin D*
7:00	2.03	263.44	1.42	184.59	0.03	4.45	0.02	2.93
8:00	3.12	404.71	3.08	400.01	0.38	49.40	0.44	56.98
9:00	4.69	609.00	4.78	620.00	0.98	126.51	1.13	145.95
10:00	6.22	806.69	6.28	814.58	1.27	164.30	1.46	189.41
11:00	6.95	901.56	7.48	970.03	1.30	168.59	1.25	162.16
12:00	7.17	929.58	7.98	1034.93	1.37	177.28	1.41	182.98
13:00	6.36	824.77	8.03	1041.57	1.47	191.05	1.64	213.08
14:00	4.50	584.14	5.59	724.68	0.94	122.37	0.84	109.14
15:00	3.00	389.50	3.84	498.64	0.26	33.52	0.23	29.47
16:00	2.10	271.77	2.41	312.06	0.05	7.03	0.05	6.68
Average	4.61	598.52	5.09	660.11	0.81	104.45	0.85	109.88

## Data Availability

Data sharing not applicable.

## References

[1] World Health Organization (1994). Ultraviolet Radiation, Environmental Health Criteria 160.

[2] Reichrath J. (2006). The challenge resulting from positive and negative effects of sunlight: How much solar UV exposure is appropriate to balance between risks of vitamin D deficiency and skin cancer?. Prog. Biophys. Mol. Biol..

[3] Brustad M., Edvardsen K., Wilsgaard T., Engelsen O., Aksnes L., Lund E. (2007). Seasonality of UV-radiation and vitamin D status at 69 degrees north. Photochem. Photobiol. Sci..

[4] Holick M.F. (2004). Sunlight and vitamin D for bone health and prevention of autoimmune diseases, cancers, and cardiovascular disease. Am. J. Clin. Nutr..

[5] Engelsen O. (2010). The relationship between ultraviolet radiation exposure and vitamin D status. Nutrients.

[6] Choi E.Y. (2012). 25 (OH) D status and demographic and lifestyle determinants of 25 (OH) D among Korean adults. Asia Pac. J. Clin. Nutr..

[7] Serrano M.A., Cañada J., Moreno J.C., Gurrea G. (2017). Solar ultraviolet doses and vitamin D in a northern mid-latitude. Sci. Total Environ..

[8] Kim H.S., Oh S.T., Lim J.H. (2018). Development of local area alert system against particulate matters and ultraviolet rays based on open IoT platform with P2P. Peer-to-Peer Netw. Appl..

[9] Herndon J.M., Hoisington R.D., Whiteside M. (2018). Deadly ultraviolet UV-C and UV-B penetration to Earth’s surface: Human and environmental health implications. J. Geog. Environ. Earth Sci. Int..

[10] Banerjee S., Hoch E.G., Kaplan P.D., Dumont E.L. A comparative study of wearable ultraviolet radiometers. Proceedings of the 2017 IEEE Life Sciences Conference (LSC).

[11] Ghosh A., Riccardi G. Recognizing human activities from smartphone sensor signals. Proceedings of the 22nd ACM international conference on Multimedia.

[12] Kim Y.S., Kwon S.Y., Lim J.H. (2016). Implementation of light quality evaluation system using smartphone. Int. J. Bio-Sci. Bio-Technol..

[13] Salman A.G., Kanigoro B., Heryadi Y. Weather forecasting using deep learning techniques. Proceedings of the 2015 International Conference on Advanced Computer Science and Information Systems (ICACSIS).

[14] Kazanasmaz T., Günaydin M., Binol S. (2009). Artificial neural networks to predict daylight illuminance in office buildings. Build. Environ..

[15] Mei B., Li R., Cheng W., Yu J., Cheng X. (2017). Ultraviolet radiation measurement via smart devices. IEEE Int. Things J..

[16] Feister U., Laschewski G., Grewe R.D. (2011). UV index forecasts and measurements of health-effective radiation. J. Photochem. Photobiol. B: Biol..

[17] Şahin F., Işik G., Şahin G., Kara M.K. (2020). Estimation of PM10 levels using feed forward neural networks in Igdir, Turkey. Urban Clim..

[18] Jacovides C.P., Tymvios F.S., Boland J., Tsitouri M. (2015). Artificial Neural Network models for estimating daily solar global UV, PAR and broadband radiant fluxes in an eastern Mediterranean site. Atmos. Res..

[19] Afifi M., Brown M.S. (2019). Sensor-independent illumination estimation for DNN models. arXiv.

[20] Allaart M., van Weele M., Fortuin P., Kelder H. (2004). An empirical model to predict the UV-index based on solar zenith angles and total ozone. Meteorol. Appl..

[21] Park D.H., Oh S.T., Lim J.H. (2019). Development of a UV index sensor-based portable measurement device with the EUVB ratio of natural light. Sensors.

[22] Park S.K., Moon H.J., Min K.C., Hwang C., Kim S. (2018). Application of a multiple linear regression and an artificial neural network model for the heating performance analysis and hourly prediction of a large-scale ground source heat pump system. Energy Build..

[23] Kriegeskorte N., Golan T. (2019). Neural network models and deep learning. Curr. Biol..

[24] Saez Y., Baldominos A., Isasi P. (2017). A comparison study of classifier algorithms for cross-person physical activity recognition. Sensors.

[25] Merenda M., Porcaro C., Iero D. (2020). Edge Machine Learning for AI-enabled IoT devices: A review. Sensors.

[26] Freeman B.S., Taylor G., Gharabaghi B., Thé J. (2018). Forecasting air quality time series using deep learning. J. Air Waste Manag. Assoc..

[27] Kök İ., Şimşek M.U., Özdemir S. A deep learning model for air quality prediction in smart cities. Proceedings of the 2017 IEEE International Conference on Big Data (Big Data).

[28] Russo A., Raischel F., Lind P.G. (2013). Air quality prediction using optimal neural networks with stochastic variables. Atmos. Environ..

[29] Stathakis D. (2009). How many hidden layers and nodes?. Int. J. Remote Sens..

[30] Potdar K., Pardawala T.S., Pai C.D. (2017). A comparative study of categorical variable encoding techniques for neural network classifiers. IJCA.

[31] Arora R., Basu A., Mianjy P., Mukherjee A. (2016). Understanding deep neural networks with rectified linear units. arXiv.

[32] Manning J., Langerman D., Ramesh B., Gretok E., Wilson C., George A., MacKinnon J., Crum G. Machine-learning space applications on smallsat platforms with tensorflow. Proceedings of the 32nd Annual AIAA/USU Conference on Small Satellites.

[33] Godar D.E., Pope S.J., Grant W.B., Holick M.F. (2012). Solar UV Doses of Young Americans and Vitamin D3 Production. Environ. Health Perspect..

[34] Hettiaratchy S., Papini R. (2004). Initial management of a major burn: II—assessment and resuscitation. Br. Med. J..

[35] Godar D.E., Pope S.J., Grant W.B., Holick M.F. (2011). Solar UV doses of adult americans and vitamin D 3 production. Dermatoendocrinol.

[36] Pope S.J., Holick M.F., Mackin S., Godar D.E. (2008). Action spectrum conversion factors that change erythemally weighted to previtamin D3-weighted UV doses. Photochem. Photobiol..

[37] Pope S.J., Godar D.E. (2010). Solar UV geometric conversion factors: Horizontal plane to cylinder model. Photochem. Photobiol..

